# A Prospectively Validated Nomogram for Predicting the Risk of PHQ-9 Score ≥15 in Patients With Erectile Dysfunction: A Multi-Center Study

**DOI:** 10.3389/fpubh.2022.836898

**Published:** 2022-06-17

**Authors:** Yu Zheng, Ming Gao, Guangdong Hou, Niuniu Hou, Xiao Feng, Tommaso B. Jannini, Di Wei, Wanxiang Zheng, Lei Zhang, Xinlong Dun, Geng Zhang, Fuli Wang, Ping Meng, Emmanuele A. Jannini, Jianlin Yuan

**Affiliations:** ^1^Department of Urology, Xijing Hospital, Air Force Medical University, Xi'an, China; ^2^Department of Anatomy, Histology and Embryology, Air Force Medical University, Xi'an, China; ^3^Medical Innovation Center, Air Force Medical University, Xi'an, China; ^4^Department of Andrology, Xi'an Daxing Hospital, Shaanxi University of Chinese Medicine, Xi'an, China; ^5^Department of General Surgery, Eastern Theater Air Force Hospital of PLA, Nanjing, China; ^6^Department of Systems Medicine, University of Rome Tor Vergata, Rome, Italy

**Keywords:** erectile dysfunction, major depression, nomogram, prognostic factor, risk factor

## Abstract

**Background:**

Although erectile dysfunction (ED) often occurs simultaneously with depression, not all patients with ED suffer major depression (MD), with a PHQ-9 score ≥15 indicating MD. Because the PHQ-9 questionnaire includes phrases such as “I think I am a loser” and “I want to commit suicide,” the psychological burdens of ED patients are likely to increase inevitably after using the PHQ-9, which, in turn, may affect ED therapeutic effects. Accordingly, we endeavored to develop a nomogram to predict individual risk of PHQ-9 score ≥15 in these patients.

**Methods:**

The data of 1,142 patients with ED diagnosed in Xijing Hospital and Northwest Women and Children's Hospital from January 2017 to May 2020 were analyzed. While the Least Absolute Shrinkage and Selection Operator regression was employed to screen PHQ-9 score ≥15 related risk factors, multivariate logistic regression analysis was performed to verify these factors and construct the nomogram. The training cohort and an independent cohort that comprised 877 prospectively enrolled patients were used to demonstrate the efficacy of the nomogram.

**Results:**

The IIEF-5 score, PEDT score, physical pain score, frequent urination, and feeling of endless urination were found to be independent factors of PHQ-9 score ≥15 in patients with ED. The nomogram developed by these five factors showed good calibration and discrimination in internal and external validation, with a predictive accuracy of 0.757 and 0.722, respectively. The sensitivity and specificity of the nomogram in the training cohort were 0.86 and 0.52, respectively. Besides, the sensitivity and specificity of the nomogram in the validation cohort were 0.73 and 0.62, respectively. Moreover, based on the nomogram, the sample was divided into low-risk and high-risk groups.

**Conclusion:**

This study established a nomogram to predict individual risk of PHQ-9 score ≥15 in patients with ED. It is deemed that the nomogram may be employed initially to avoid those with a low risk of MD completing questionnaires unnecessarily.

## Introduction

Erectile dysfunction (ED), which is one of the most common andrological diseases, refers to the inability of men to obtain and maintain sufficient erections continuously to complete satisfactory sexual intercourse. ED mostly affects men over the age of 40 years and has a considerable incidence throughout the world. The EAU 2021 Andrology Disease Guide notes that the incidence of ED, which ranges from 12 to 82.9%, increases with age (1). An epidemiological survey in China revealed that the comprehensive prevalence of ED is as high as 49.69%. Furthermore, 20.86, 25.3, 40.48, 60.12, 79.1, and 93.72% of men aged under 30 years, between 30 and 39, 40 and 49, 50 and 59, 60 and 69, and over 70 years, respectively suffer ED (2). Although ED is not life-threatening, it may leave those affected feeling inferior, embarrassed, anxious, and depressed, which may have an adverse effect on their quality of life.

It is well established in the literature that major depression (MD) is consistently associated with sexual dysfunction (SD). Indeed, SD may be the result of either the pathophysiology of the disease itself or the psychopharmacotherapy patients with MD frequently undergo (3). Among sexual impairments, absent or delayed orgasm, premature ejaculation (PE), decreased libido, difficulties with arousal, and ED are listed as the most common ones (4). Although ED is one of the most common male diseases, which often occurs in conjunction with depression, the “chicken-and-egg relationship” between ED and depression remains unclear (5). Some studies have shown that both conditions share a common pathophysiological basis, with the one affecting the other (6–9). Shiri et al. (10) found that the incidence of ED was 59/1,000 person-years (95% confidence interval [CI] 39–90) in men with depressive mood and 37/1,000 person-years (95% CI 32–43) in those free of the disorder. Moreover, a recent systematic review and meta-analysis reported pooled odds ratio (OR) for risk of ED among patients with MD was 1.39 (11). For these reasons, it is imperative that patients with MD should be routinely screened for ED, and, on the contrary, patients with ED must be routinely screened for MD (12). To this end, the psychological evaluation of patients with ED is of paramount importance for two reasons. Firstly, patients with ED usually experience PE, which, in turn, is a risk factor for depression (13); secondly, the first-line PE treatment selective serotonin reuptake inhibitors (SSRIs) may frequently concur to produce ED while treating ejaculation impairments (14).

There are many scales used to screen for depression, such as the 9-item version of the Patient Health Questionnaires (PHQ-9), Beck Depression Inventory-II (BDI), Center for Epidemiological Studies-Depression Scale-20 (CES-D), Common Mental Disorder Questionnaireare (CMDQ) and so on (15). Among these methods, PHQ-9 has been reported to be an easy-to-use and the most reliable one, with a total score of ≥15 considered as an MD candidate (16). However, routine clinical practice shows that the psychological burdens of ED patients may increase more or less after using the PHQ-9 because it includes phrases such as “I think I am a loser” and “I want to commit suicide”. In turn, these PHQ-9-induced psychological burdens may have a negative impact on ED therapy. Therefore, we endeavored to develop a nomogram to predict individual risk of PHQ-9 score ≥15 in patients with ED, so that the treatment outcomes of patients with low risk of PHQ-9 score ≥15, who should be provided with close follow-up rather than the PHQ-9, may be improved.

## Materials and Methods

### Study Sample

**Figure 1:**
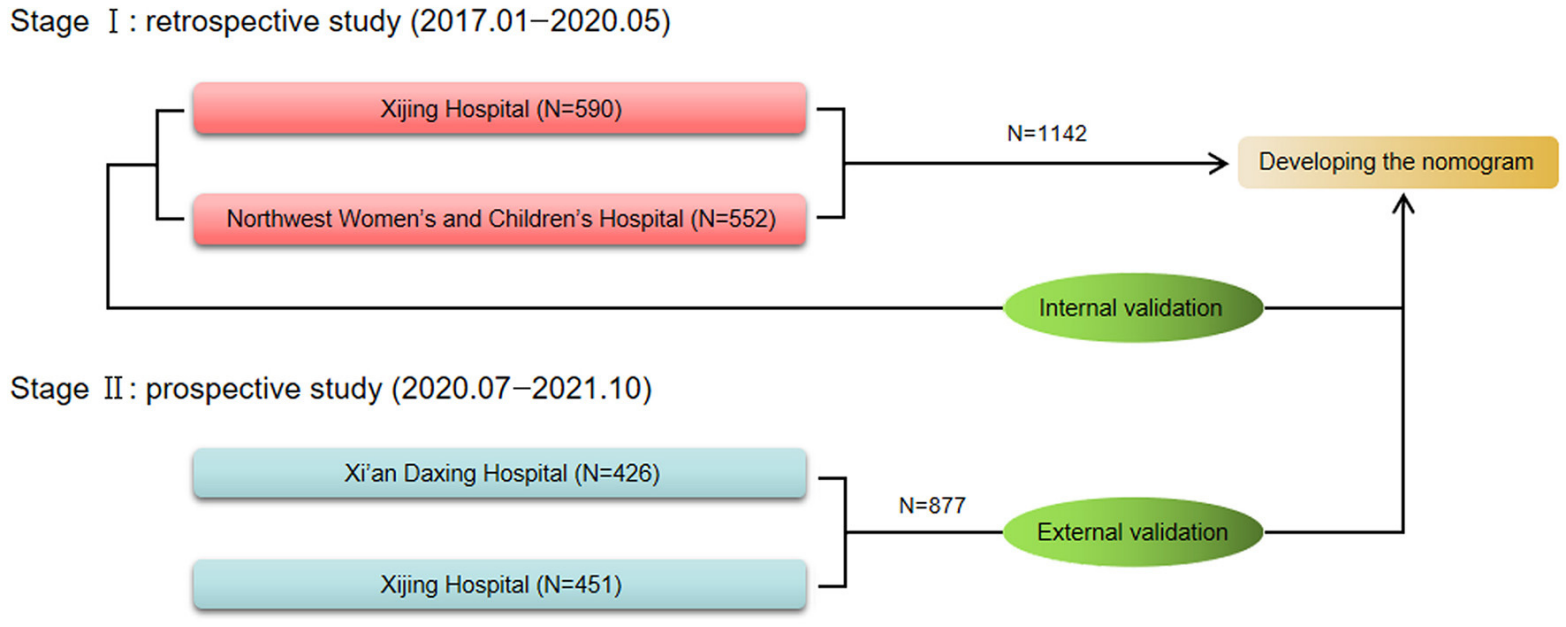
The flow chart of this study.

The training cohort comprised 1,142 patients with ED diagnosed at Xijing Hospital (Xi'an, China) and Northwest Women and Children's Hospital (Xi'an, China) from January 2017 to May 2020. The data of the patients in this cohort were retrospectively analyzed to explore independent predictors of MD and develop the nomogram. After the completion and internal validation of the nomogram, the data of patients with ED diagnosed in Daxing hospital (Xi'an, China) and Xijing Hospital (Xi'an, China) from July 2020 to November 2021 were collected prospectively. Finally, the data of 877 patients were collected and employed as an independent verification cohort for external verification of the nomogram. The flowchart of this study is depicted in Figure 1.

The inclusion and exclusion criteria of all the patients in the cohort that established and verified the model are as follows. The patients had to be at least 18 years old, involved in a monogamous sexual relationship and have had vaginal intercourse for at least 2 months, have had sexual intercourse at least once a month during the previous 2 months, not taken drugs such as antidepressants and type 5 phosphodiesterase inhibitors that may have affected their ability to have an erection during the previous 2 months, not suffer from any other serious neurological and/or mental disorders, and ensure that all information provided was correct. Two experienced urologists employed the diagnostic criteria of the International Society of Sexual Medicine (ISSM) (17) to diagnose ED. The PHQ-9 was employed to assess MD and as noted previously, a total score of ≥15 was considered MD candidate (13). All the patients who participated in the study provided written informed consent. Furthermore, all the procedures performed in our study were approved by the ethics committee of Xijing Hospital (No. KY20212177-F-1).

### Variable Selection

The variables included in this study were age at visit, residence (town/city or countryside), income, years of education, the 5-item International Index of Erectile Function (IIEF-5) questionnaire score (18), premature ejaculation diagnostic tool (PEDT) score (19), frequency of sexual desire, feeling of endless urination, and frequent urination, that is, having to urinate within 2 h of urinating during the previous 2 weeks. In addition, their average physical body pain intensity during the previous 2 weeks was evaluated by employing the short form of the Brief Pain Inventory (20), which assesses pain on a numerical scale, ranging from 0 (no pain) to 10 (the most severe pain imaginable). According to the IIEF-5 score, erectile function is divided into four grades: without ED (≥22), mild ED (12–21), moderate ED (8–11), and severe ED (5–7).

### Statistical Analysis

The Kolmogorov Smirnov test demonstrated the discrete variables and only continuous variable, namely, age at visit to be a nonnormal distribution as frequency (proportion) and median (interquartile range), respectively. While the distribution of discrete variables between the training and validation cohorts was evaluated by means of a chi square test, the Mann–Whitney *U*-test was employed to assess the distribution of age at visit. The Least Absolute Shrinkage and Selection Operator (LASSO) (21) was adopted to reduce the dimensionality of the data and prevent the model from overfitting (22). The nomogram was developed using R software (version 4.0.3) by integrating independent predictors identified by multivariable logistic regression models. In the internal verification, the calibration chart of the nomogram was drawn by comparing prediction and observation. The discrimination of the nomogram was reflected by the area under the curve (AUC) of the receiver operating characteristic (ROC) (23), ranging from 0.5 (equivalent to tossing a coin) to 1 (implying excellent discrimination). Furthermore, 1,000-times-repeated bootstrapping resampling was performed for these evaluations. The sensitivity and specificity of the nomogram were identified according to the maximum Youden index on the basis of the ROC curve. Similarly, in the external verification, a calibration chart was drawn, and AUC was calculated to verify the performance of the nomogram further. Moreover, restricted cubic splines analyses based on logistic regression models with five knots (at the 5th, 27.5th, 50th, 72.5th, and 95th percentiles) of the continuous total points were performed to assess the exposure-response relationship between the predictive point and MD (24).

Two-tailed *P* values <0.05 were considered to be statistically significant.

## Results

### Characteristics of Patients and Variables

The proportion of PHQ-9 score ≥15 in the training cohort and validation cohort are 12.4% (142/1,142) and 11.6% (102/877), respectively. The characteristics of the two cohorts are displayed in Table 1. No statistical difference between the two cohorts was found in the comparison of the 10 basic variables (all *P* > 0.05; Table 1). Besides, the PHQ-9 scores in the two cohorts are 7 (4–11) and 7 (4–11), respectively (*P* = 0.859). In the training cohort, patients from the western, central, and eastern regions of China accounted for 57.8% (660/1,142), 26.5% (303/1,142), and 15.7% (179/1,142), respectively, and the proportions in the validation cohort were 51.4% (451/877), 27.7% (243/877), and 20.9% (183/877), respectively.

**Table 1:** Comparison of variables between training cohort and validation cohort.

**Variables**	**Training cohort** **(*N* = 1,142)**	**Validation cohort** **(*N* = 877)**	***P*-Value**
**Age at visit (years)**, ***n*** **(%)**		
≤ 25	196 (17.2)	137 (15.6)	0.617
26–30	413 (36.2)	320 (36.5)	
31–35	307 (26.9)	229 (26.1)	
>35	226 (19.8)	191 (21.8)	
**Residence**, ***n*** **(%)**		
town/city	864 (75.7)	682 (77.8)	0.268
countryside	278 (24.3)	195 (22.2)	
**Income (RMB/month)**, ***n*** **(%)**
<5,000	787 (68.9)	573 (65.3)	0.236
5,000–10,000	273 (23.9)	234 (26.7)	
>10,000	82 (7.2)	70 (8.0)	
**Years of education**, ***n*** **(%)**		
Years ≤ 9	229 (20.1)	168 (19.2)	0.637
9 < years <16	532 (46.6)	399 (45.5)	
Years ≥16	381 (33.4)	310 (35.3)	
**IIEF-5 score**, ***n*** **(%)**		
5–7	73 (6.4)	59 (6.7)	0.965
8–11	204 (17.9)	155 (17.7)	
12–16	406 (35.6)	304 (34.7)	
17–21	459 (40.2)	359 (40.9)	
**PEDT score**, ***n*** **(%)**		
≤ 8	181 (15.8)	162 (18.5)	0.400
9–10	104 (9.1)	77 (8.8)	
11–14	348 (30.5)	271 (30.9)	
≥15	509 (44.6)	367 (41.8)	
**Physical pain score**, ***n*** **(%)**		
0	613 (53.7)	494 (56.3)	0.611
1	316 (27.7)	220 (25.1)	
2	81 (7.1)	67 (7.6)	
3	65 (5.7)	43 (4.9)	
≥4	67 (5.9)	53 (6.0)	
**Frequency of sexual desire**, ***n*** **(%)**		
Hardly any	281 (24.6)	201 (22.9)	0.550
A few times	358 (31.3)	282 (32.2)	
About half the time	441 (38.6)	335 (38.2)	
Most of the time	62 (5.4)	59 (6.7)	
**Frequent urination**, ***n*** **(%)**
Hardly any	720 (63.0)	546 (62.3)	0.730
A few times	158 (13.8)	136 (15.5)	
About half the time	129 (11.3)	92 (10.5)	
Most of the time	135 (11.8)	103 (11.7)	
**Feeling of endless urination**, ***n*** **(%)**
Hardly any	773 (67.7)	593 (67.6)	0.732
A few times	138 (12.1)	103 (11.7)	
About half the time	74 (6.5)	49 (5.6)	
Most of the time	157 (13.7)	132 (15.1)	

*IIEF-5, 5-item International Index Erectile Function; PEDT, premature ejaculation diagnostic tool.*

### The Selection of Predictors and the Construction of the Nomogram

**Figure 2:**
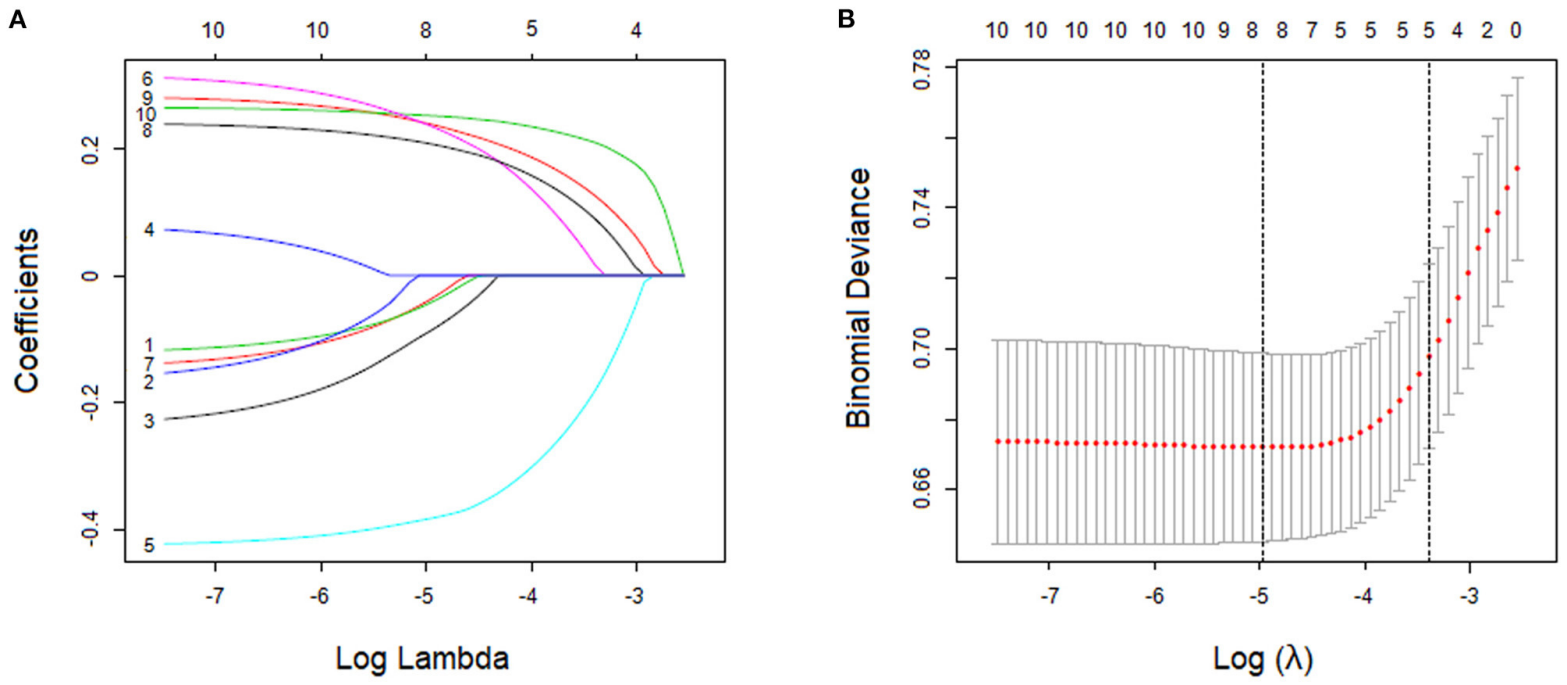
Variable screening using the least absolute shrinkage and selection operator (LASSO) binary logistic regression model. **(A)** LASSO coefficient profile of features. This graph shows the relationship between the penalty coefficient log (λ) and the retained charicteristics. The number of intersecting curves represent the number of charicteristics retained at that log (λ) value. **(B)** The relationship between log (λ) and binomial deviance. Based on the 10-fold crossvalidation method, the relationship between log (λ) and binomial deviance is drawn. The dotted vertical lines were drawn at the optimal values by using the minimum criteria and the minimum criterion of 1 SE.

With nonzero coefficients in the LASSO regression, five potential predictors (IIEF-5 score, PEDT score, physical pain score, frequent urination, and feeling of endless urination) were selected from the 10 variables included in the study (Figures 2A,B). The AUCs of the IIEF-5 score, PEDT score, physical pain score, frequent urination, and feeling of endless urination in the training cohort were 0.639, 0.605, 0.631, 0.658, and 0.667, respectively. Furthermore, the AUCs of these variables in the validation cohort were 0.625, 0.610, 0.626, 0.613, and 0.632, respectively.

**Figure 3:**
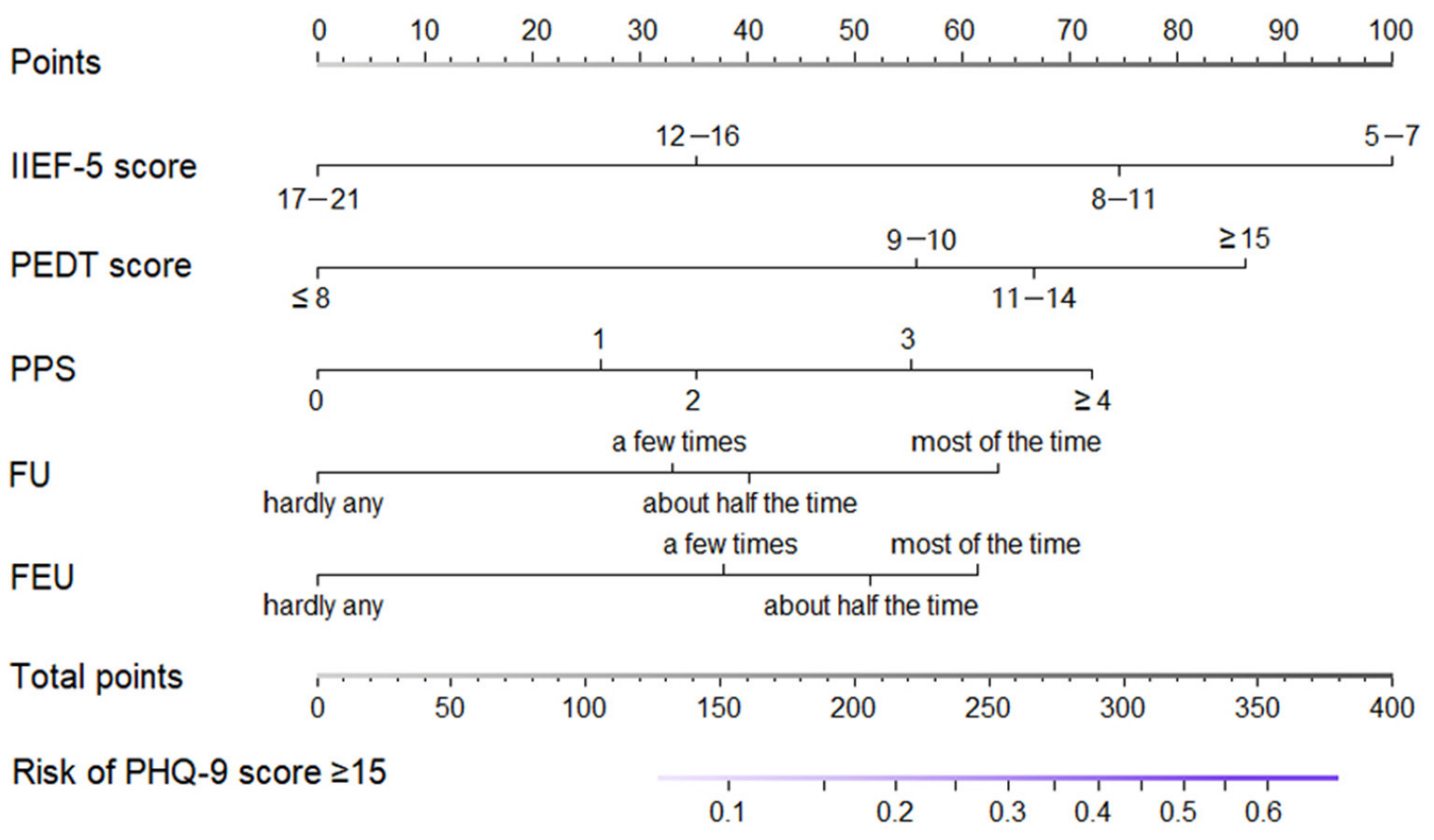
Nomogram for predicting individual risk of major depression in patients with erectile dysfunction. (IIEF-5, 5-item International Index Erectile Function; PEDT, premature ejaculation diagnostic tool; PPS, physical pain score; FU, frequent urination; FEU, feeling of endless urination).

All the five potential predictors selected by the LASSO regression were independent predictors when the multivariate logistic regression analysis was performed. All odd ratio and *P* values are presented in Table 2. R software was employed to integrate these five independent predictors and develop the nomogram in accordance with the regression coefficients of these predictor variables (Figure 3).

**Table 2:** Multivariable logistic regression of predictors for screening major depression in patients with erectile dysfunction (training cohort).

**Variables**	**OR**	**95% CI**	**P value**
**IIEF-5 score**			<0.001
17–21	1 (reference)		
12–16	1.582	0.974–2.569	0.064
8–11	2.637	1.558–4.463	<0.001
5–7	3.669	1.864–7.221	<0.001
**PEDT score**			0.023
≤ 8	1 (reference)		
9–10	2.065	0.783–5.449	0.143
11–14	2.377	1.094–5.165	0.029
≥15	3.070	1.469–6.414	0.003
**Physical pain score**			0.041
0	1 (reference)		
1	1.409	0.886–2.238	0.147
2	1.580	0.803–3.108	0.185
3	2.049	1.037–4.049	0.039
≥4	2.552	1.319–4.936	0.005
**Frequent urination**			0.038
Hardly any	1 (reference)		
A few times	1.538	0.891–2.656	0.122
About half the time	1.686	0.934–3.044	0.083
Most of the time	2.277	1.275–4.068	0.005
**Feeling of endless urination**			0.026
Hardly any	1 (reference)		
A few times	1.636	0.927–2.888	0.090
About half the time	1.951	0.986–3.862	0.055
Most of the time	2.223	1.275–3.876	0.005

*CI, confidence interval; IIEF-5, 5-item International Index Erectile Function; OR, odd ratio;*

*PEDT, premature ejaculation diagnostic tool*.

### Evaluation of the Nomogram

**Figure 4:**
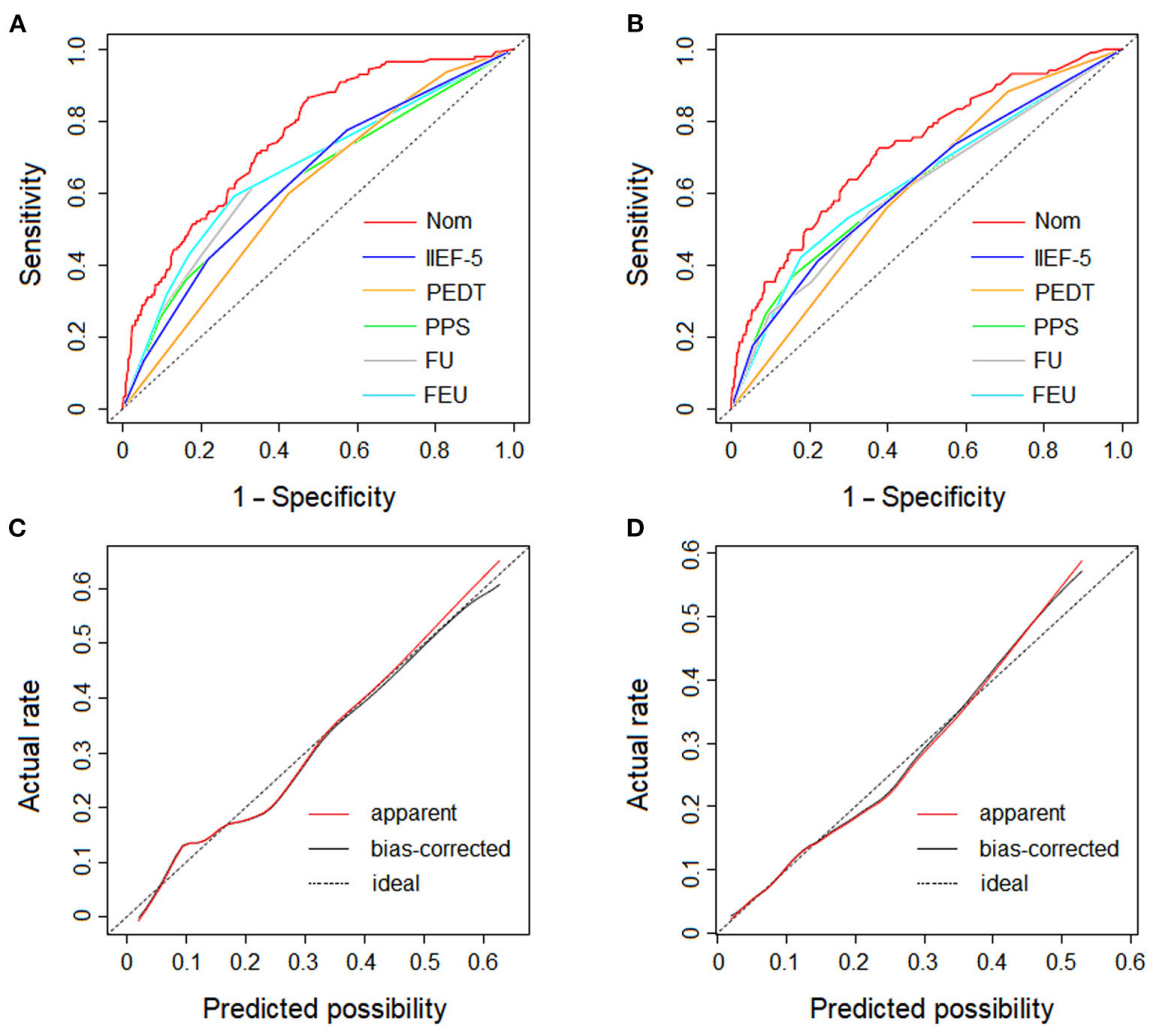
The ROC curves and the dotted line represents of the derivation cohort and validation cohort. **(A)** The ROC curves of the nomogram, IIEF-5 score, PEDT score, physical pain score, frequent urination and feeling of endless urination in derivation cohort. **(B)** The ROC curves of the nomogram, IIEF-5 score, PEDT score, physical pain score, frequent urination and feeling of endless urination in validation cohort. **(C)** The calibration plot of the nomogram in derivation cohort. **(D)** The calibration plot of the nomogram in validation cohort. The dotted line represents the calibration of an ideal nomogram (the predicted risk perfectly corresponds to the actual rate). The red solid line represents the apparent accuracy of the nomogram without correction for overfitting, while the black solid line represents the bootstrap-corrected nomogram. (Nom, nomogram; IIEF-5, 5-item International Index Erectile Function; PEDT, premature ejaculation diagnostic tool; PPS, physical pain score; FU, frequent urination; FEU, feeling of endless urination).

In the training cohort, the AUC of the nomogram was 0.757 (95% CI: 0.754–0.760), which proves the mid-to-high discrimination ability thereof. In addition, the AUC of the nomogram was statistically higher than the IIEF-5 (0.757 vs. 0.639, *P* <0.001), PEDT (0.757 vs. 0.605, *P* < 0.001), physical pain score (0.757 vs. 0.631, *P* < 0.001), frequent urination (0.757 vs. 0.658, *P* < 0.001), and feeling of endless urination (0.757 vs. 0.667, *P* < 0.001; Figure 4A). Similarly, in the validation cohort, the nomogram AUC was 0.722 (95% CI: 0.718–0.726), which also demonstrates an AUC higher than other factors such as the IIEF-5 (0.722 vs. 0.625, *P* < 0.001), PEDT (0.722 vs. 0.610, *P* < 0.001), physical pain score (0.722 vs. 0.626, *P* < 0.001), frequent urination (0.722 vs. 0.613, *P* < 0.001), and feeling of endless urination (0.722 vs. 0.632, *P* < 0.001; Figure 4B). Additionally, the sensitivity and specificity of the nomogram in the training cohort are 0.86 and 0.52, respectively; and the sensitivity and specificity in the validation cohort are 0.73 and 0.62, respectively. In the training cohort, good consistency between the predicted possibility and actual rate was observed (Figure 4C). Furthermore, in the validation cohort, excellent calibration of the nomogram was realized (Figure 4D).

### Dose-Response Analysis

**Figure 5:**
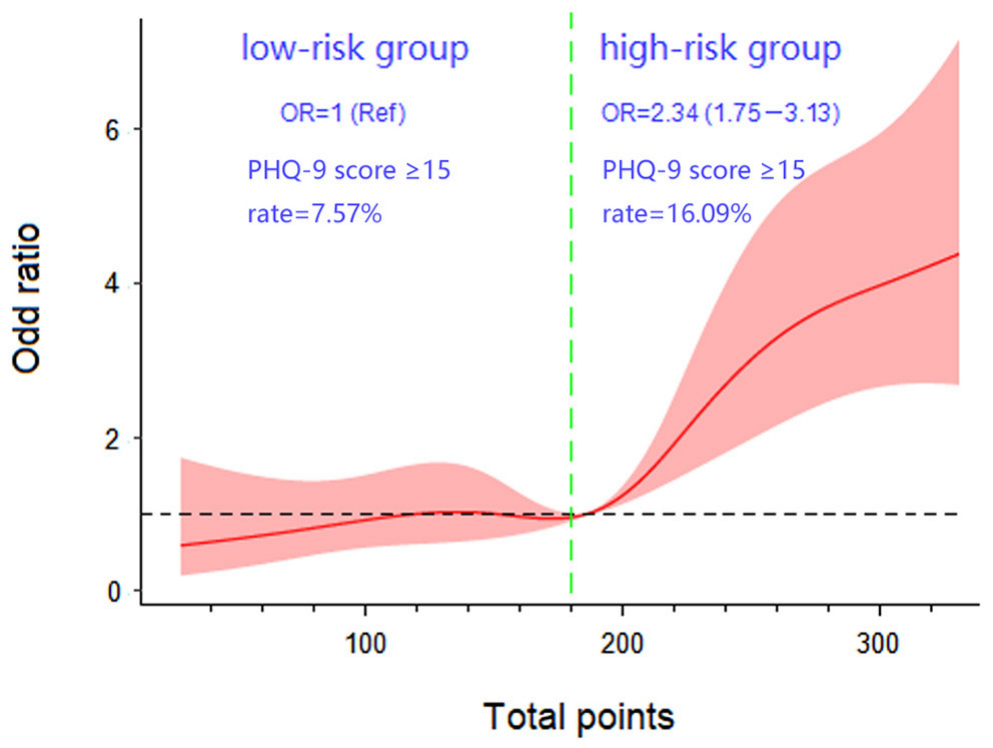
Dose-response association between total points. The presence of major depression in patients with erectile dysfunction with five knots located at the 5th, 27.5th, 50th, 72.5th, and 95th percentiles. (Ref, reference; OR, odds ratio).

Dose-response analysis demonstrated a nonlinear relationship between the nomogram-predicted total points and PHQ-9 score ≥15 (overall association *P* < 0.001, nonlinear *P* = 0.030). The risk of PHQ-9 score ≥15 was relatively flat until approximately 180 points of the total-points level and then started to increase rapidly (*P* for nonlinearity <0.05). Therefore, 180 points was identified as a reference according to the trend of the restricted cubic spline and accordingly, the participants were divided into low-risk and high-risk groups. The PHQ-9 score ≥15 rates of the two groups were 7.57 and 16.09%, respectively. The overall risk of MD in the high-risk group was 2.34 (95% CI 1.75–3.13) times higher than that of low-risk group (Figure 5).

## Discussion

The PHQ-9 is the most reliable tool for screening depression. But it may carry the risk of having adverse side effects on patients with ED who tend to worry that their condition will advance as described in the scale. Therefore, it is necessary to develop a nomogram to predict individual risk of PHQ-9 score ≥15 before using the PHQ-9, which may help clinical decision making.

The nomogram confirmed that the IIEF-5 score and PEDT score were independent risk factors for MD. First, the IIEF-5 score and PEDT score reflect the state of male sexual life jointly and depression has been found to be related to a decrease of sexual activity in male patients (25). Second, as observed in clinical practice, PE often occurs simultaneously with ED, thus resulting in a vicious circle, recently described as Loss of Control over Erection and Ejaculation (LCEE) (26). When a man with ED tries to achieve or maintain an erection through strong stimulation, his precoital excitement may be higher than that of normal healthy men, which results in shorter intravaginal ejaculation latency time. Similarly, when a patient with PE attempts to control ejaculation, his level of sex excitement decreases instinctively, which may lead to ED (27, 28). Finally, SSRIs used in the treatment of PE and MD may cause ED (3). Depending on the various drugs, the incidence of ED may range from 25.8 to 80.3% (14). Unfortunately, ED may persist after SSRIs are discontinued, with this treacherous condition being only recently defined as post-SSRI sexual dysfunction (PSSD) (29, 30).

Physical pain is another independent risk factor for MD. Research has revealed that between 30 and 60% of patients who suffer chronic pain have a depressive state (31), and more than 60% of patients with MD experience physical pain (32). This suggests that somatic pain and MD are closely related. Its mechanisms may include a high overlap of brain regions involved in depression and pain (33) because they share a common underlying brain network (34); Furthermore, inflammatory inducible factors such as Interleukin-1B may induce depression-like behavior and cause hyperalgesia. Finally, the HPA axis may be involved in the interaction between depression and pain (35).

Frequent urination and feeling of endless urination may be classified in the category of lower urinary tract symptoms (LUTS). Research throughout the world has shown that LUTS are related to ED and depression (36–38). Frequent urination and feeling of endless urination are also independent risk factors for MD in our nomogram. The common pathophysiological mechanisms of LUTS and ED include changes in the nitric oxide/cGMP pathway in the prostate and penis, rho kinase activation, endothelial pathway regulation, autonomic overactive pathways, and changes secondary to pelvic atherosclerosis (39). The correlation between LUTS and depression is long-established, with the first one being associated with a poor quality of life and therefore leading to depressive symptoms (40). Furthermore, also a reduction of serotonin in the brain has been documented. This neurochemical change in the central nervous system that may is well-known to be associated with depression may also affect the autonomic nervous activity that regulates the urinary tract, thus resulting in LTUS (41).

This work suffers from a number limitations. Firstly, although it is a multicenter study, data were collected from only three centers. Despite the three sampling centers being located in Xi'an, Shaanxi Province, China, only 29.7% (339/1,142) and 30.1% (267/877) of the two cohorts were from Shaanxi Province. However, the sample includes most areas in China and therefore, has a certain degree of representativeness. Secondly, the IIEF-5 and PEDT scales in the study are self-assessment scales, which may have led to an overestimation of the results because of the subjective nature thereof.

## Conclusions

In this study, a nomogram that can predict the risk of patients with ED of being comorbid with PHQ-9 score ≥15 was developed, and the stability of the nomogram was verified through an independent cohort. According to the nomogram, patients with ED can be divided into a low-risk group and high-risk group. While the first had an PHQ-9 score ≥15 rate of 7.57%, that of the second one was 16.09%. Hence, it is recommended that patients in the high-risk group complete the PHQ-9 and those in the low-risk group are monitored closely. This may avoid the potential adverse effects patients with a low risk may suffer when completing the PHQ-9. It is recommended that the nomogram should be evaluated on a large sample.

## Data Availability Statement

The original contributions presented in the study are included in the article/supplementary material, further inquiries can be directed to the corresponding author.

## Ethics Statement

All the procedures performed in our study were approved by the ethics committee of Xijing Hospital (No. KY20212177-F-1). The patients/participants provided their written informed consent to participate in this study.

## Author Contributions

Concept and design: JY, EJ, YZ, and GH. Acquisition, analysis, or interpretation of data: MG, GH, XF, YZ, LZ, and XD. Drafting of the manuscript: YZ, GH, MG, and NH. Critical revision of the manuscript: EJ, JY, NH, and TJ. Statistical analysis: DW, WZ, and GH. Obtained funding: JY, EJ, and MG. Administrative, technical, or material support: GZ, PM, and FW. Supervision: JY and EJ. All authors contributed to the article and approved the submitted version.

## Funding

This study was financially supported by the National Natural Science Foundation of China (No. 61971425), Italian Ministry of University PRIN (No. 2017S9KTNE_002), Youth Fund Project of National Natural Science Foundation of China (No. 62001370), China Postdoctoral Science Foundation Grant (No. 2019M650985).

## Conflict of Interest

EJ has been a consultant and/or paid speaker for Bayer, Ibsa, Lundbeck, Menarini, Otsuka, Pfizer, and Shionogi. The remaining authors declare that the research was conducted in the absence of any commercial or financial relationships that could be construed as a potential conflict of interest.

## Publisher's Note

All claims expressed in this article are solely those of the authors and do not necessarily represent those of their affiliated organizations, or those of the publisher, the editors and the reviewers. Any product that may be evaluated in this article, or claim that may be made by its manufacturer, is not guaranteed or endorsed by the publisher.
